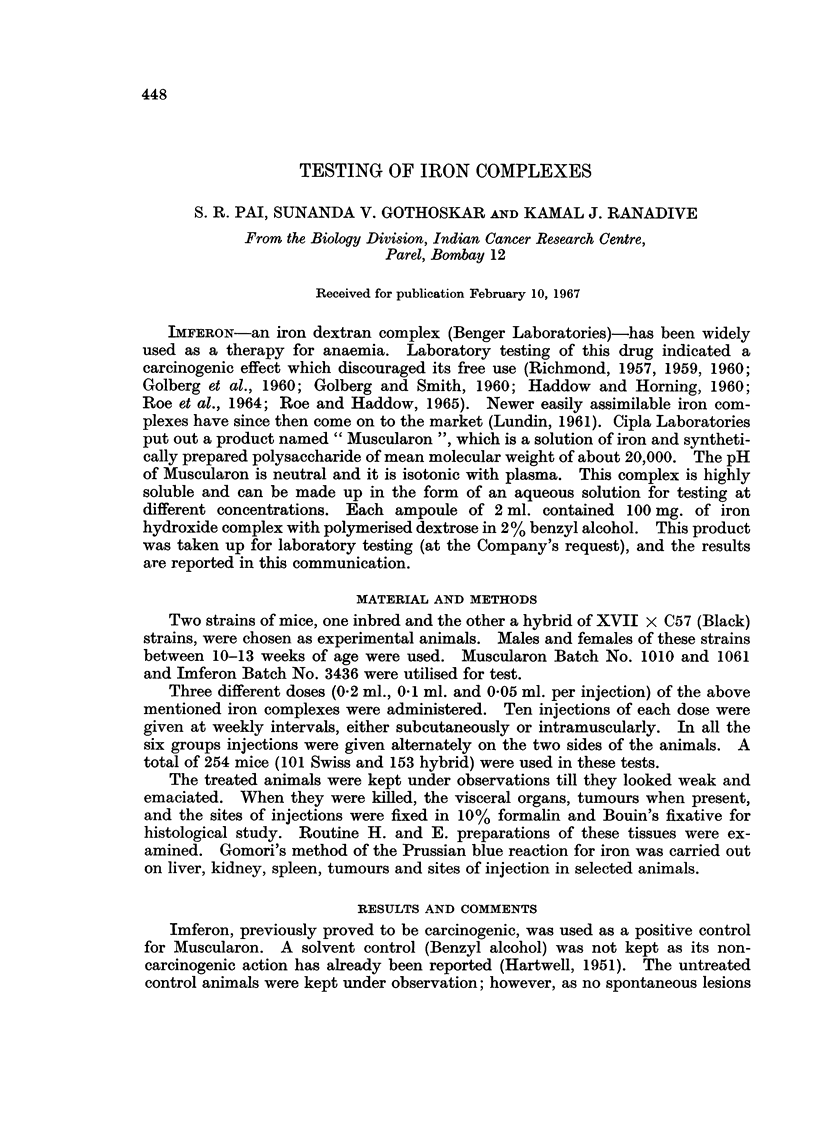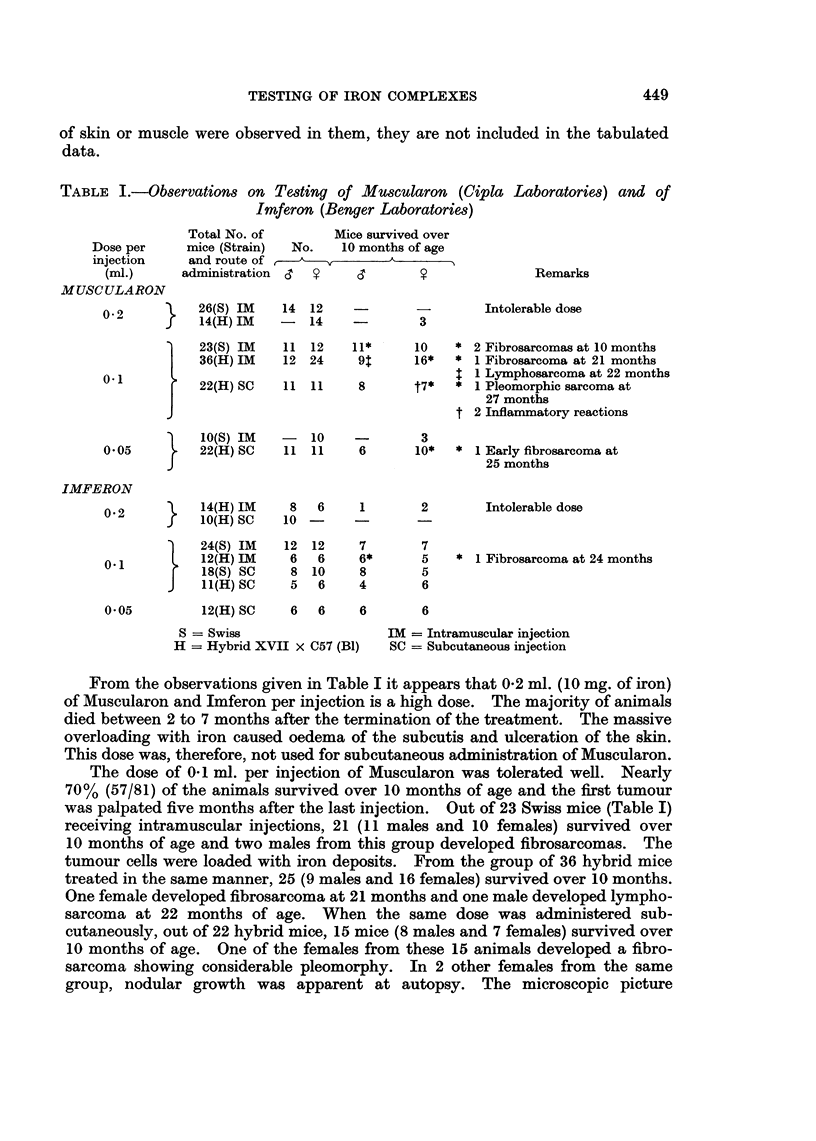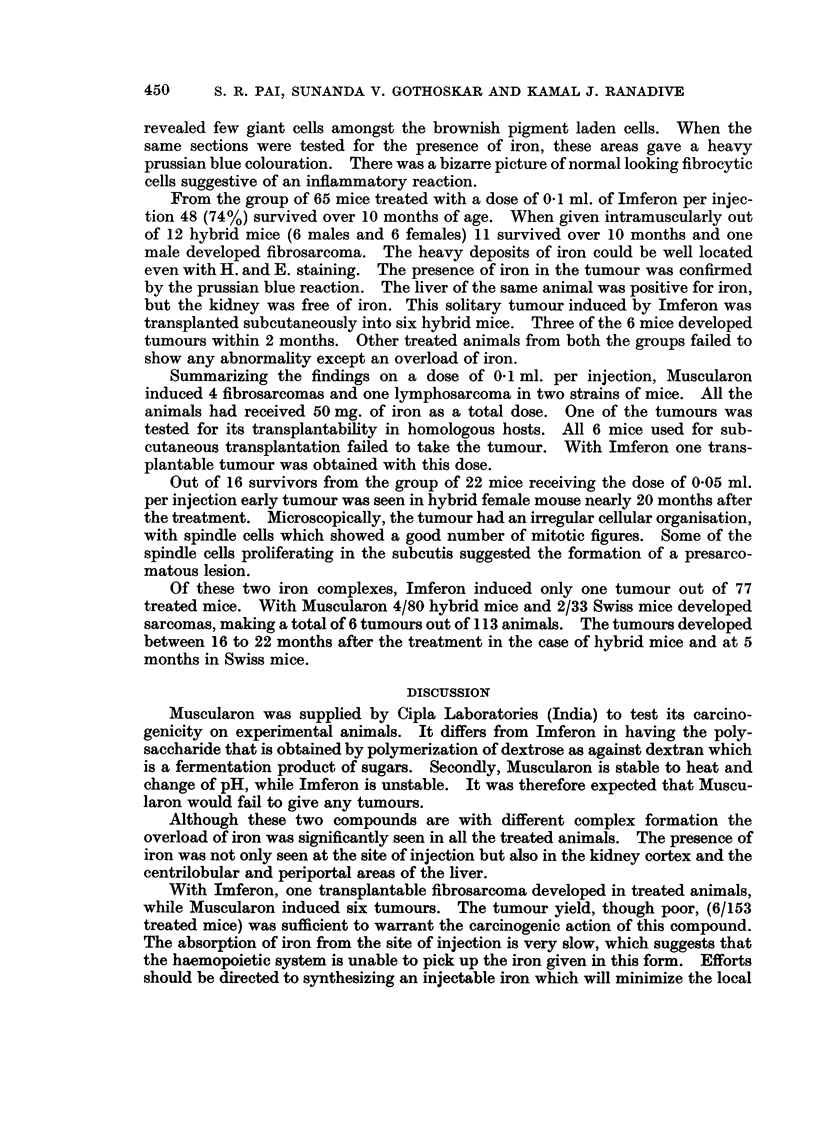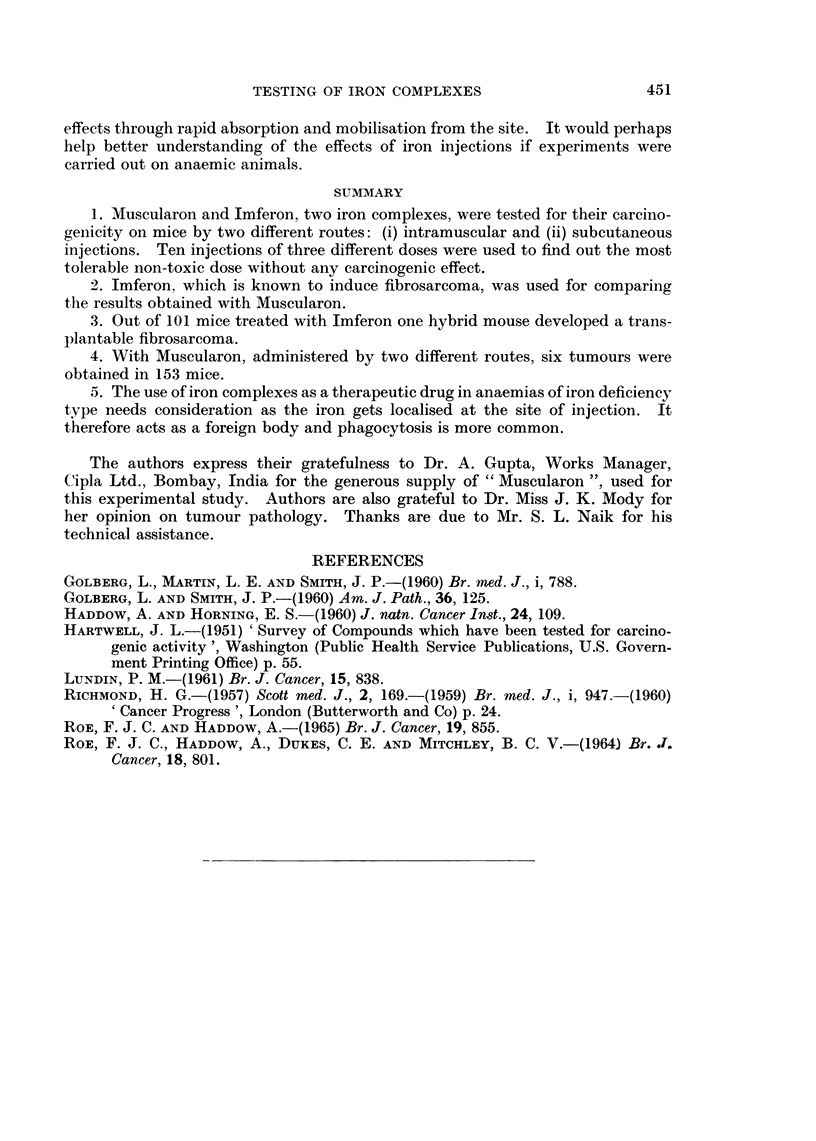# Testing of iron complexes.

**DOI:** 10.1038/bjc.1967.51

**Published:** 1967-06

**Authors:** S. R. Pai, S. V. Gothoskar, K. J. Ranadive


### TESTING OF IRON COMPLEXES

S. R. PAI, SUNANDA V. GOTHOSKAR AND KAMAL J. RANADIVE

*From the Biology Division, Indian Cancer Research Centre, Parel, Bombay 12*

Received for publication February 10, 1967

IMFERON—an iron dextran complex (Benger Laboratories)—has been widely used as a therapy for anaemia. Laboratory testing of this drug indicated a carcinogenic effect which discouraged its free use (Richmond, 1957, 1959, 1960; Golberg *et al.*, 1960; Golberg and Smith, 1960; Haddow and Horning, 1960; Roe *et al.*, 1964; Roe and Haddow, 1965). Newer easily assimilable iron complexes have since then come on to the market (Lundin, 1961). Cipla Laboratories put out a product named " Muscularon ", which is a solution of iron and synthetically prepared polysaccharide of mean molecular weight of about 20,000. The pH of Muscularon is neutral and it is isotonic with plasma. This complex is highly soluble and can be made up in the form of an aqueous solution for testing at different concentrations. Each ampoule of 2 ml. contained 100 mg. of iron hydroxide complex with polymerised dextrose in 2% benzyl alcohol. This product was taken up for laboratory testing (at the Company's request), and the results are reported in this communication.

### MATERIAL AND METHODS

Two strains of mice, one inbred and the other a hybrid of XVII × C57 (Black) strains, were chosen as experimental animals. Males and females of these strains between 10–13 weeks of age were used. Muscularon Batch No. 1010 and 1061 and Imferon Batch No. 3436 were utilised for test.

Three different doses (0·2 ml., 0·1 ml. and 0·05 ml. per injection) of the above mentioned iron complexes were administered. Ten injections of each dose were given at weekly intervals, either subcutaneously or intramuscularly. In all the six groups injections were given alternately on the two sides of the animals. A total of 254 mice (101 Swiss and 153 hybrid) were used in these tests.

The treated animals were kept under observations till they looked weak and emaciated. When they were killed, the visceral organs, tumours when present, and the sites of injections were fixed in 10% formalin and Bouin's fixative for histological study. Routine H. and E. preparations of these tissues were examined. Gomori's method of the Prussian blue reaction for iron was carried out on liver, kidney, spleen, tumours and sites of injection in selected animals.

### RESULTS AND COMMENTS

Imferon, previously proved to be carcinogenic, was used as a positive control for Muscularon. A solvent control (Benzyl alcohol) was not kept as its non-carcinogenic action has already been reported (Hartwell, 1951). The untreated control animals were kept under observation; however, as no spontaneous lesions of skin or muscle were observed in them, they are not included in the tabulated data.

TABLE I.—*Observations on Testing of Muscularon (Cipla Laboratories) and of Imferon (Benger Laboratories)*

| Dose per injection (ml.) | Total No. of mice (Strain) and route of administration | No. ♂ | No. ♀ | Mice survived over 10 months of age ♂ | Mice survived over 10 months of age ♀ | Remarks |
|---|---|---|---|---|---|---|
| *MUSCULARON* | | | | | | |
| 0·2 | 26(S) IM | 14 | 12 | — | — | Intolerable dose |
| | 14(H) IM | — | 14 | — | 3 | |
| 0·1 | 23(S) IM | 11 | 12 | 11* | 10 | * 2 Fibrosarcomas at 10 months |
| | 36(H) IM | 12 | 24 | 9‡ | 16* | * 1 Fibrosarcoma at 21 months<br>‡ 1 Lymphosarcoma at 22 months |
| | 22(H) SC | 11 | 11 | 8 | †7* | * 1 Pleomorphic sarcoma at 27 months<br>† 2 Inflammatory reactions |
| 0·05 | 10(S) IM | — | 10 | — | 3 | |
| | 22(H) SC | 11 | 11 | 6 | 10* | * 1 Early fibrosarcoma at 25 months |
| *IMFERON* | | | | | | |
| 0·2 | 14(H) IM | 8 | 6 | 1 | 2 | Intolerable dose |
| | 10(H) SC | 10 | — | — | — | |
| 0·1 | 24(S) IM | 12 | 12 | 7 | 7 | |
| | 12(H) IM | 6 | 6 | 6* | 5 | * 1 Fibrosarcoma at 24 months |
| | 18(S) SC | 8 | 10 | 8 | 5 | |
| | 11(H) SC | 5 | 6 | 4 | 6 | |
| 0·05 | 12(H) SC | 6 | 6 | 6 | 6 | |

S = Swiss IM = Intramuscular injection

H = Hybrid XVII × C57 (Bl) SC = Subcutaneous injection

From the observations given in Table I it appears that 0·2 ml. (10 mg. of iron) of Muscularon and Imferon per injection is a high dose. The majority of animals died between 2 to 7 months after the termination of the treatment. The massive overloading with iron caused oedema of the subcutis and ulceration of the skin. This dose was, therefore, not used for subcutaneous administration of Muscularon.

The dose of 0·1 ml. per injection of Muscularon was tolerated well. Nearly 70% (57/81) of the animals survived over 10 months of age and the first tumour was palpated five months after the last injection. Out of 23 Swiss mice (Table I) receiving intramuscular injections, 21 (11 males and 10 females) survived over 10 months of age and two males from this group developed fibrosarcomas. The tumour cells were loaded with iron deposits. From the group of 36 hybrid mice treated in the same manner, 25 (9 males and 16 females) survived over 10 months. One female developed fibrosarcoma at 21 months and one male developed lymphosarcoma at 22 months of age. When the same dose was administered subcutaneously, out of 22 hybrid mice, 15 mice (8 males and 7 females) survived over 10 months of age. One of the females from these 15 animals developed a fibrosarcoma showing considerable pleomorphy. In 2 other females from the same group, nodular growth was apparent at autopsy. The microscopic picture revealed few giant cells amongst the brownish pigment laden cells. When the same sections were tested for the presence of iron, these areas gave a heavy prussian blue colouration. There was a bizarre picture of normal looking fibrocytic cells suggestive of an inflammatory reaction.

From the group of 65 mice treated with a dose of 0·1 ml. of Imferon per injection 48 (74%) survived over 10 months of age. When given intramuscularly out of 12 hybrid mice (6 males and 6 females) 11 survived over 10 months and one male developed fibrosarcoma. The heavy deposits of iron could be well located even with H. and E. staining. The presence of iron in the tumour was confirmed by the prussian blue reaction. The liver of the same animal was positive for iron, but the kidney was free of iron. This solitary tumour induced by Imferon was transplanted subcutaneously into six hybrid mice. Three of the 6 mice developed tumours within 2 months. Other treated animals from both the groups failed to show any abnormality except an overload of iron.

Summarizing the findings on a dose of 0·1 ml. per injection, Muscularon induced 4 fibrosarcomas and one lymphosarcoma in two strains of mice. All the animals had received 50 mg. of iron as a total dose. One of the tumours was tested for its transplantability in homologous hosts. All 6 mice used for subcutaneous transplantation failed to take the tumour. With Imferon one transplantable tumour was obtained with this dose.

Out of 16 survivors from the group of 22 mice receiving the dose of 0·05 ml. per injection early tumour was seen in hybrid female mouse nearly 20 months after the treatment. Microscopically, the tumour had an irregular cellular organisation, with spindle cells which showed a good number of mitotic figures. Some of the spindle cells proliferating in the subcutis suggested the formation of a presarcomatous lesion.

Of these two iron complexes, Imferon induced only one tumour out of 77 treated mice. With Muscularon 4/80 hybrid mice and 2/33 Swiss mice developed sarcomas, making a total of 6 tumours out of 113 animals. The tumours developed between 16 to 22 months after the treatment in the case of hybrid mice and at 5 months in Swiss mice.

### DISCUSSION

Muscularon was supplied by Cipla Laboratories (India) to test its carcinogenicity on experimental animals. It differs from Imferon in having the polysaccharide that is obtained by polymerization of dextrose as against dextran which is a fermentation product of sugars. Secondly, Muscularon is stable to heat and change of pH, while Imferon is unstable. It was therefore expected that Muscularon would fail to give any tumours.

Although these two compounds are with different complex formation the overload of iron was significantly seen in all the treated animals. The presence of iron was not only seen at the site of injection but also in the kidney cortex and the centrilobular and periportal areas of the liver.

With Imferon, one transplantable fibrosarcoma developed in treated animals, while Muscularon induced six tumours. The tumour yield, though poor, (6/153 treated mice) was sufficient to warrant the carcinogenic action of this compound. The absorption of iron from the site of injection is very slow, which suggests that the haemopoietic system is unable to pick up the iron given in this form. Efforts should be directed to synthesizing an injectable iron which will minimize the local effects through rapid absorption and mobilisation from the site. It would perhaps help better understanding of the effects of iron injections if experiments were carried out on anaemic animals.

SUMMARY

1. Muscularon and Imferon, two iron complexes, were tested for their carcinogenicity on mice by two different routes: (i) intramuscular and (ii) subcutaneous injections. Ten injections of three different doses were used to find out the most tolerable non-toxic dose without any carcinogenic effect.

2. Imferon, which is known to induce fibrosarcoma, was used for comparing the results obtained with Muscularon.

3. Out of 101 mice treated with Imferon one hybrid mouse developed a transplantable fibrosarcoma.

4. With Muscularon, administered by two different routes, six tumours were obtained in 153 mice.

5. The use of iron complexes as a therapeutic drug in anaemias of iron deficiency type needs consideration as the iron gets localised at the site of injection. It therefore acts as a foreign body and phagocytosis is more common.

The authors express their gratefulness to Dr. A. Gupta, Works Manager, Cipla Ltd., Bombay, India for the generous supply of " Muscularon ", used for this experimental study. Authors are also grateful to Dr. Miss J. K. Mody for her opinion on tumour pathology. Thanks are due to Mr. S. L. Naik for his technical assistance.

REFERENCES

Golberg, L., Martin, L. E. and Smith, J. P.—(1960) *Br. med. J.*, i, 788.

Golberg, L. and Smith, J. P.—(1960) *Am. J. Path.*, **36**, 125.

Haddow, A. and Horning, E. S.—(1960) *J. natn. Cancer Inst.*, **24**, 109.

Hartwell, J. L.—(1951) ' Survey of Compounds which have been tested for carcinogenic activity ', Washington (Public Health Service Publications, U.S. Government Printing Office) p. 55.

Lundin, P. M.—(1961) *Br. J. Cancer*, **15**, 838.

Richmond, H. G.—(1957) *Scott med. J.*, **2**, 169.—(1959) *Br. med. J.*, i, 947.—(1960) ' Cancer Progress ', London (Butterworth and Co) p. 24.

Roe, F. J. C. and Haddow, A.—(1965) *Br. J. Cancer*, **19**, 855.

Roe, F. J. C., Haddow, A., Dukes, C. E. and Mitchley, B. C. V.—(1964) *Br. J. Cancer*, **18**, 801.